# Oral, Vaginal, and Placental Microbiota Profiles in Japanese Pregnancies with Preterm Birth and Chronic Abruption-Oligohydramnios Sequence (CAOS): A Cross-Sectional Study

**DOI:** 10.3390/medicina61122096

**Published:** 2025-11-25

**Authors:** Yuka Fukuma, Yoshifumi Kasuga, Keisuke Akita, Junko Tamai, Yuya Tanaka, Keita Hasegawa, Toshimitsu Otani, Marie Fukutake, Satoru Ikenoue, Satoru Morikawa, Taneaki Nakagawa, Kazuyuki Ishihara, Mamoru Tanaka

**Affiliations:** 1 Department of Obstetrics and Gynecology, Keio University School of Medicine, 35 Shinanomachi, Shinjuku-ku, Tokyo 160-8582, Japan; 2 Department of Dentistry and Oral Surgery, Keio University School of Medicine, 35 Shinanomachi, Shinjuku-ku, Tokyo 160-8582, Japan; 3 Department of Microbiology, Tokyo Dental College, 2-9-18 Kandamisaki-cho, Chiyoda-ku, Tokyo 101-0061, Japan

**Keywords:** microbiota, preterm birth, chronic abruption-oligohydramnios sequence, vaginal dysbiosis, 16S rRNA gene sequencing

## Abstract

*Background and Objectives*: Preterm birth (PTB) imposes a substantial medical and economic burden on perinatal care. Recent advances in 16S rRNA gene sequencing help detailed microbiota analysis. Understanding microbiota’s contribution may help in understanding PTB pathogenesis. We aim to investigate the microbiota profiles of the oral, vaginal, and placental microbiota in pregnant Japanese women hospitalized for care of preterm labor and examine the association between them and perinatal outcomes. *Materials and Methods*: This cross-sectional study included 20 pregnant Japanese women admitted to a single perinatal center for preterm labor between 2022 and 2023. Oral, vaginal, and placental samples were collected aseptically during hospitalization. The patients were retrospectively categorized into: term birth (TB, *n* = 10), chronic abruption-oligohydramnios sequence (CAOS, *n* = 3), and PTB without CAOS (PTB, *n* = 7) perinatal outcomes. Microbiota profiles were analyzed using 16S rRNA gene sequencing, and group comparisons were performed using univariate statistical methods. *Results*: Alpha or beta diversity of the oral and vaginal microbiota among the three groups did not differ significantly. CAOS and PTB groups showed a trend toward altered vaginal microbial composition, but not the TB group. In the placental microbiota, beta diversity differed significantly among the TB, PTB, and CAOS groups. *Ureaplasma urealyticum* was more abundant in the PTB group, whereas *Ureaplasma parvum* was more abundant in the CAOS group. *Conclusions*: A potential shift in the vaginal microbiota and alterations in the placental microbiota, observed in PTB, including CAOS, suggested a possible microbial contribution.

## 1. Introduction

In 2020, an estimated 13.4 million babies were born preterm, defined as birth before 37 weeks of gestation [1]. Preterm birth (PTB) occurs in 11% of births worldwide and 6% in Japan [2], and PTB rates have not significantly declined in any region over the past decade [1]. PTB is the leading cause of death in children below five [3], and requires not only pregnancy management but also long-term offspring care, resulting in enormous medical costs. Preventing PTB is a critical global health priority.

Several factors contribute to PTB, and risk factors can be categorized into those present before conception, those specific to the current pregnancy, and infection-related factors [3,4]. Preconception factors include pregnancy within 12 months, a history of PTB, family history of PTB, prior cervical conization or radial trachelectomy, smoking, low socioeconomic status, adolescent pregnancy, and underweight (BMI < 18.5 kg/m^2^) [3,4,5,6,7,8,9,10]. Current pregnancy-related factors include shortened cervical length, multi-fetal gestation, anemia, early pregnancy bleeding, and male fetal sex [3,4,11,12,13,14,15]. Infection-related factors include bacterial vaginosis, asymptomatic bacteriuria, and periodontal disease [16,17,18]. Furthermore, chronic abruption-oligohydramnios sequence (CAOS) is a serious obstetric complication contributing to PTB, characterized by poor perinatal outcomes due to placental dysfunction, fetal growth restriction, and oligohydramnios [19,20,21]. A Japanese multicenter study reported mean delivery at 25.2 ± 2.8 gestational age with poor neonatal outcomes, often death or chronic lung disease [22]. In CAOS, affected infants often experience more severe respiratory complications than preterm infants of similar gestational ages [23]. Nevertheless, only a limited number of CAOS cases have been reported globally, and its pathophysiology remains unclear. Clinicians have also suggested a possible association between CAOS and chorioamnionitis (CAM), highlighting the urgent need for further elucidation.

Evidence strongly supports infection as a major contributor to many PTB cases [24]. Recent advances in 16S rRNA sequencing, amplifying conserved bacterial sequences via polymerase chain reaction (PCR) followed by next-generation sequencing, have enabled detailed analysis of the microbiota. Regarding the vaginal microbiota, prior studies consistently show that *Lactobacillus crispatus*-dominant vaginal microbiota has been associated with a lower risk of spontaneous PTB, whereas low-*Lactobacillus* community status or high diversity has been linked to increased risk [25,26,27,28]. In contrast, findings regarding the roles of the oral and placental microbiota in PTB remain inconsistent [29,30], and the existence of a placental microbiota is still a subject of debate [31,32].

This pilot study aimed to investigate the association between the oral, vaginal, and placental microbiota and PTB in pregnant women hospitalized for preterm labor. It provides new data on the microbiota across multiple maternal sites in PTB, including rare CAOS cases with the first microbiota analysis worldwide.

## 2. Materials and Methods

### 2.1. Study Design

This cross-sectional study was conducted under a protocol approved by the ethics committee of our University (approval number: 20211075). This study adhered to the STROBE guidelines. Between February 2022 and February 2023, 20 pregnant women hospitalized for preterm labor were enrolled after providing written informed consent. Multi-fetal pregnancies, fetal anomalies, and placenta previa were excluded. None of the participants received progesterone therapy or underwent cervical cerclage during the study period. Preterm labor was defined as regular uterine contractions, vaginal bleeding, or shortening of the cervical length calculated by transvaginal ultrasound between 22 weeks 0 days and 36 weeks 6 days of gestation [33]. The expected delivery date was calculated by crown–rump length at 10–11 weeks of gestation using ultrasound.

### 2.2. Study Participants

All 20 participants were Japanese women. Ten participants delivered prematurely. Among the PTBs, three cases met the criteria for CAOS, a condition characterized by chronic genital bleeding and oligohydramnios [19]. CAOS diagnosis was based on fulfillment of all three criteria: (1) clinically significant vaginal bleeding in the absence of placenta previa or other identifiable source of bleeding, (2) amniotic fluid volume initially documented as normal, and (3) oligohydramnios (amniotic fluid index ≦ 5) eventually developing without concurrent evidence of ruptured membranes. The study cohort included ten patients who delivered at term (TB group), seven who delivered preterm (PTB group), and three diagnosed with CAOS (CAOS group).

### 2.3. Procedures

Following hospital admission, oral samples were self-collected under clinician supervision, while vaginal samples were obtained by a clinician. Placental tissue was collected at delivery. Using sterile instruments, a tissue fragment was excised from the inner parenchymal region, avoiding contact with the maternal or fetal surfaces. Sampling was performed approximately 1 cm away from the membranes, without surface decontamination. All samples were immediately placed in the microbiome kit, sealed, and stored at −20 °C until DNA extraction.

An OMNIgene ORAL or VAGINAL microbiome kit (KYODO INTERNATIONAL Inc., Kawasaki, Japan) containing swab tips and DNA/RNA-stabilizing liquid tubes was used. The swabbing movement involved tracing several full circles along the oral, vaginal, and placental walls for 20 s. Thereafter, the swab was immediately inserted into a collection tube containing a stabilizing liquid for the microbiota. The apex of the swab tip was placed in a liquid tube. The samples were immediately transferred to Varinos Inc., Tokyo, Japan, where the oral, vaginal, and placental microbiota were analyzed using 16S rRNA sequencing. One participant in the PTB group declined to provide vaginal samples, and as a result, the vaginal sample sizes were TB: *n* = 10, PTB: *n* = 6, and CAOS: *n* = 3.

### 2.4. 16S rRNA Gene Sequencing and Microbiota Profiling

Genomic DNA was extracted using the MagNA Pure 24 System (Pathogen 1000 hp 3.1 protocol). The V1–V2 region of the 16S rRNA gene was amplified using primers 27Fmod and 338R with Illumina overhang adaptors. Sterile distilled water processed identically to the samples was used as negative controls, with 1–2 controls included in each sequencing run. These controls showed very low read counts and bacterial profiles clearly distinct from the placental samples, indicating minimal contamination. Potential contaminants were assessed using the decontam package, but we did not rely solely on this approach; samples with fewer than approximately 1000 effective reads after background removal were considered environmental noise and were excluded from downstream analyses. Sequencing libraries were prepared according to the Illumina 16S Metagenomic Sequencing Library Preparation protocol and sequenced on a MiSeq platform with 251 bp paired-end reads. Sequence data were quality-filtered and clustered into amplicon sequence variant (ASVs) using QIIME 2 [34]. Taxonomic assignment was performed against a custom database based on SILVA138. A detailed protocol is described in Appendix A.

### 2.5. Statistical Analysis

The primary endpoints were the α-diversity and β-diversity analyses for each microbiota. Continuous variables were compared using the Kruskal–Wallis rank-sum test or Student’s *t*-test, and categorical data using the chi-squared test or Fisher’s exact test. A *p*-value < 0.05 was considered statistically significant. Statistical analyses for alpha diversity and beta diversity among groups were carried out by Kruskal–Wallis and permutational multivariate analysis of variance (PERMANOVA, 999 permutations) [35], respectively, using QIIME 2. False discovery rate (FDR) correction for pairwise taxon comparisons was applied using the Benjamini–Hochberg method.

## 3. Results

Maternal characteristics and perinatal outcomes were compared among the three groups, revealing significant differences in maternal age, elective or emergency cesarean section rates, gestational age at placental sample collection, and birth weight (Table 1).

### 3.1. Oral Microbiota

**Figure 1:**
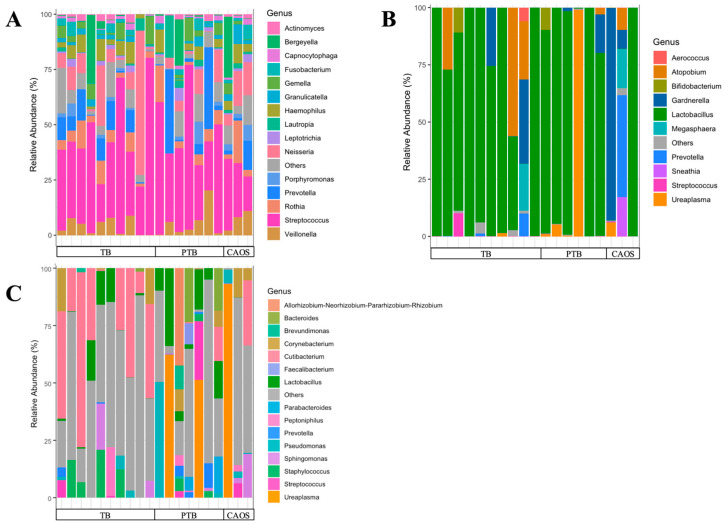
Oral, vaginal, and placental microbial community structures in term birth (TB), preterm birth (PTB), and CAOS groups. Stacked bar plots show the relative abundance (%) of bacterial genera in each sample, grouped from the term birth (TB), preterm birth (PTB), and CAOS groups. (**A**) Oral microbiota composition showing the top 15 genera (TB: *n* = 10, PTB: *n* = 7, CAOS: *n* = 3). (**B**) Vaginal microbiota composition showing the top 10 genera (TB: *n* = 10, PTB: *n* = 6, CAOS: *n* = 3). (**C**) Placental microbiota composition showing the top 15 genera (TB: *n* = 10, PTB: *n* = 7, CAOS: *n* = 3). Each vertical bar represents an individual sample. Genera not included in the top taxa are categorized as “Others”.

The minimum read count was 36,899. To standardize sequencing depth, rarefaction was performed at 30,000 sequences for oral samples, followed by core metrics phylogenetic analysis (Supplementary Figure S1A). The taxonomic composition, specifically the top 15 relative abundances of the community, is shown for each individual case. Various genera were detected in each group, with *Streptococcus* being predominant (Figure 1A).

**Figure 2:**
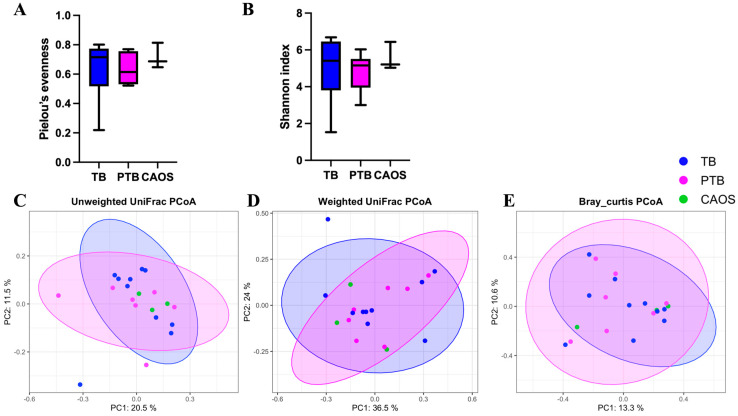
Alpha and beta diversity analyses of the oral microbiota in term birth (TB), preterm birth (PTB), and CAOS groups (TB: n = 10, PTB: n = 7, CAOS: n = 3). (**A**,**B**) Alpha diversity indices (**A**) Shannon index (TB vs. CAOS, q = 0.866; Cliff’s delta = −0.067, TB vs. PTB, q = 0.570; Cliff’s delta = 0.257, PTB vs. CAOS, q = 0.570; Cliff’s delta = −0.429), (**B**) Pielou’s evenness (TB vs. CAOS, q = 0.626; Cliff’s delta = −0.267, TB vs. PTB, q = 0.626; Cliff’s delta = 0.143, PTB vs. CAOS, q = 0.626; Cliff’s delta = −0.524). (**C**–**E**) Principal coordinates analysis (PCoA) based on (**C**) unweighted UniFrac, (**D**) weighted UniFrac, and (**E**) BrayCurtis distance matrices (PERMANOVA q = 0.906; R^2^ = 0.126, q = 0.968; R^2^ = 0.101, and q = 0.994; R^2^ = 0.114, respectively). Axes represent the first two principal coordinates, with percentages indicating the proportion of variance explained. Each point represents one sample. The ellipses represent a confidence interval of 95% around the centroid of each group cluster. q-values represent FDR-adjusted *p*-values.

Alpha diversity, assessed using Shannon and evenness indices, showed no significant differences among the TB, PTB, and CAOS groups (Shannon index: TB vs. CAOS, H = 0.286, q = 0.866; TB vs. PTB, H = 0.771, q = 0.570; PTB vs. CAOS, H = 1.052, q = 0.570. evenness index: TB vs. CAOS, H = 0.457, q = 0.626; TB vs. PTB, H = 0.238, q = 0.626; PTB vs. CAOS, H = 1.571, q = 0.626) (Figure 2A,B). Other alpha diversities were described in Supplementary Figure S2A,B.

Beta diversity was evaluated using unweighted and weighted UniFrac and Bray–Curtis distances, and no statistically significant differences were found among the groups (Figure 2C–E).

### 3.2. Vaginal Microbiota

The minimum read count was 25,618. To standardize sequencing depth, rarefaction was performed at 25,000 sequences for oral samples, followed by core metrics phylogenetic analysis (Supplementary Figure S1B). The taxonomic composition, specifically the top 10 relative abundances of the community, is shown for each individual case. Overall, Lactobacillus was dominant; however, it was not the predominant genus in CAOS cases, except in one case. In the PTB and CAOS groups, non-Lactobacillus genera such as *Gardnerella*, *Ureaplasma*, *Atopobium*, and *Prevotella* were more frequently detected (Figure 1B).

**Figure 3:**
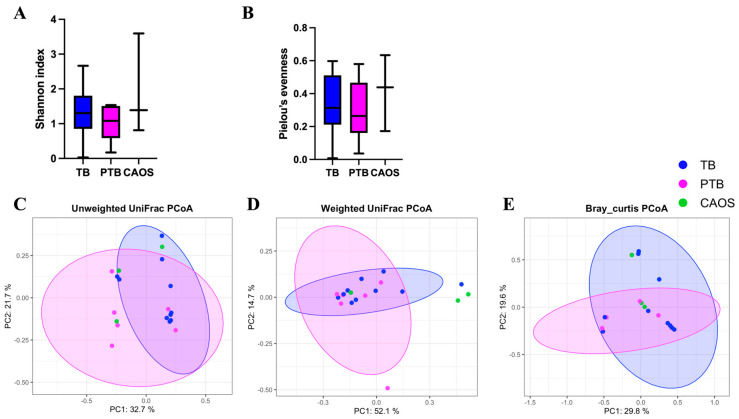
Alpha and beta diversity analyses of the vaginal microbiota in term birth (TB), preterm birth (PTB), and CAOS groups (TB: n = 10, PTB: n = 6, CAOS: n = 3). (**A**,**B**) Alpha diversity indices. (**A**) Shannon index (all q = 0.612; Cliff’s delta = TB vs. CAOS: −0.200, TB vs. PTB: 0.300, PTB vs. CAOS: −0.333), (**B**) Pielou’s evenness (all q = 0.612; Cliff’s delta = TB vs. CAOS: −0.200, TB vs. PTB: 0.167, PTB vs. CAOS: −0.333), (**C**–**E**) Principal coordinates analysis (PCoA) based on (**C**) unweighted UniFrac (TB vs. CAOS, q = 0.162; TB vs. PTB, q = 0.135; PTB vs. CAOS, q = 0.502; R^2^ = 0.081), (**D**) weighted UniFrac (TB vs. CAOS, q = 0.120; TB vs. PTB, q = 0.324; PTB vs. CAOS, q = 0.120; R^2^ = 0.069) and (**E**) Bray–Curtis distance matrices (all q = 0.313; R^2^ = 0.108). Axes represent the first two principal coordinates, with percentages indicating the proportion of variance explained. Each point represents one sample. The ellipses represent a 95% confidence interval around the centroid of each group cluster. q-values represent FDR-adjusted *p*-values.

Alpha diversity showed no significant differences among the TB, PTB, and CAOS groups (all q = 0.612) (Shannon indices: TB vs. CAOS, H = 0.257; TB vs. PTB, H = 0.953; and PTB vs. CAOS, H = 0.600). (Evenness indices: TB vs. CAOS, H = 0.257; TB vs. PTB, H = 0.294; PTB vs. CAOS, H = 0.439) (Figure 3A,B). Other alpha diversities were described in Supplementary Figure S2C,D.

Beta diversity analysis revealed no statistically significant differences among the groups after FDR correction. Unweighted UniFrac showed a statistically significant difference before correction between TB and PTB (*p* = 0.045; q = 0.135), and weighted UniFrac between PTB and CAOS (*p* = 0.046; q = 0.120). The Bray–Curtis distances showed no significant difference (q = 0.313) (Figure 3C–E).

**Figure 4:**
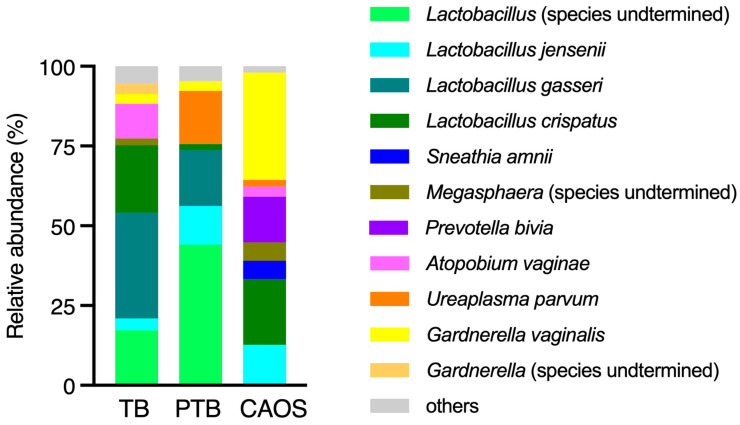
Average relative abundance of bacterial species estimated from ASV in the vaginal microbiota across the term birth (TB), preterm birth (PTB), and CAOS groups (TB: n = 10, PTB: n = 6, CAOS: n = 3). Stacked bar plots represent the mean relative abundance (%) of bacterial species detected in each group. Only species with notable abundance are shown.

The average detection rates of the bacterial species estimated from ASV in the vaginal microbiota were compared among the three groups. In the TB group, *Lactobacillus* species, such as *L. gasseri* and *L. crispatus*, were overwhelmingly dominant. In contrast, the PTB group showed an increased abundance of *Atopobium vaginae*, *Ureaplasma parvum*, and *Gardnerella vaginalis*. The CAOS group was characterized by a decreased abundance of *Lactobacillus* and a higher prevalence of anaerobes such as *Gardnerella vaginalis*, *Prevotella bivia*, *Megasphaera*, *Sneathia amnii*, and *Fusobacterium* (Figure 4).

**Figure 5:**
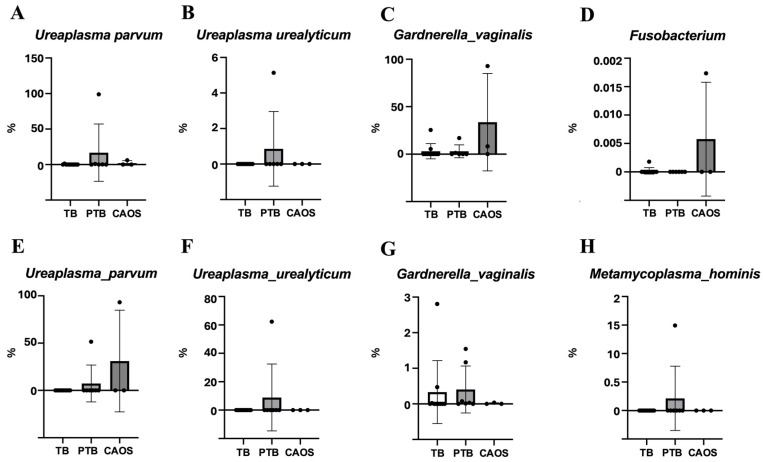
Relative abundance of specific preterm-associated bacteria in the vaginal and placental microbiota among term birth (TB), preterm birth (PTB), and CAOS groups. Box plots show the relative abundance (%) of selected bacterial species estimated from ASV previously associated with preterm birth. Each point represents an individual sample. (**A**–**D**) Vaginal microbiota (TB: *n* = 10, PTB: *n* = 6, CAOS: *n* = 3): *Ureaplasma parvum*, *Ureaplasma urealyticum*, *Gardnerella vaginalis*, and *Fusobacterium*. (**E**–**H**) Placental microbiota (TB: *n* = 10, PTB: *n* = 7, CAOS: *n* = 3): *Ureaplasma parvum*, *Ureaplasma urealyticum*, *Gardnerella vaginalis*, and *Metamycoplasma hominis* (*Mycoplasma hominis*).

The relative abundances of specific bacterial species estimated from ASV associated with PTB were analyzed in the vaginal microbiota. *Ureaplasma parvum* and *Ureaplasma urealyticum* showed higher relative abundances in the PTB group, whereas *Gardnerella vaginalis* and *Fusobacterium* were more abundant in the CAOS group (Figure 5A–D).

### 3.3. Placental Microbiota

In placental sample, four samples were removed due to low read depth to avoid biases due to sampling depth. The rarefaction cut-off was set at 2000 reads, as the alpha rarefaction curves showed that the observed species richness reached a plateau above this depth (Supplementary Figure S1C). The taxonomic composition, specifically the top 15 relative abundances of the community, is shown for each individual case. A variety of bacteria was detected across all groups, without a consistently dominant genus. One CAOS case showed a marked predominance of the genus *Ureaplasma*, accounting for 98% of the placental microbiota and 19% of the vaginal microbiota. (Figure 1C, Supplementary Figure S3).

**Figure 6:**
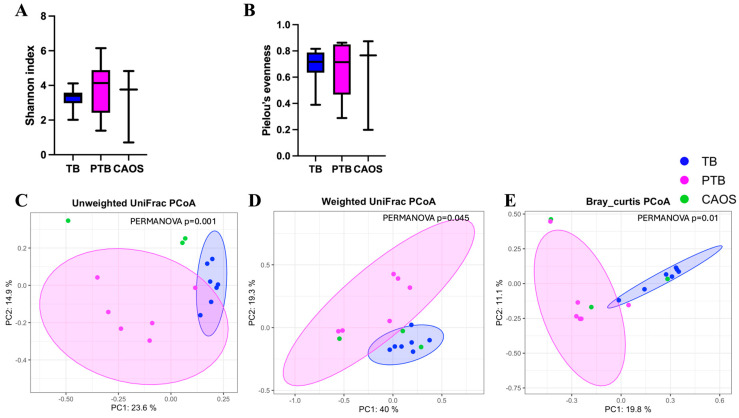
Alpha and beta diversity analyses of the placental microbiota in term birth (TB), preterm birth (PTB), and CAOS groups (TB: n = 7, PTB: n = 6, CAOS: n = 3). (**A**,**B**) Alpha diversity indices, including (**A**) Shannon index (all q = 0.606; Cliff’s delta = TB vs. CAOS: −0.238, TB vs. PTB: −0.333, PTB vs. CAOS: 0.222), (**B**) Pielou’s evenness (all q = 1.000; Cliff’s delta = TB vs. CAOS: −0.143, TB vs. PTB: −0.048, PTB vs. CAOS: 0). (**C**–**E**) Principal coordinates analysis (PCoA) based on (**C**) unweighted UniFrac (TB vs. CAOS, q = 0.012; TB vs. PTB, q = 0.009; PTB vs. CAOS, q = 0.026; R^2^ = 0.278), (**D**) weighted UniFrac (TB vs. CAOS, q = 0.465; TB vs. PTB, q = 0.006; PTB vs. CAOS, q = 0.465; R^2^ = 0.233) and (**E**) Bray–Curtis distance matrices (TB vs. CAOS, q = 0.080; TB vs. PTB, q = 0.006; PTB vs. CAOS, q = 0.630; R^2^ = 0.205). Axes represent the first two principal coordinates, with percentages indicating the proportion of variance explained. Each point represents one sample. The ellipses represent a 95% confidence interval around the centroid of each group cluster. q-values represent FDR-adjusted *p*-values.

Alpha diversity showed no statistically significant differences among the TB, PTB, and CAOS groups, although the median values were slightly higher in the PTB and CAOS groups (Shannon index: q = 0.606, TB vs. CAOS, H = 0.325; TB vs. PTB, H = 1.000; PTB vs. CAOS, H = 0.267. evenness index: q = 1.0, TB vs. CAOS, H = 0.117; TB vs. PTB, H = 0.020; PTB vs. CAOS, H = 0.000) (Figure 6A,B). Other alpha diversities were described in Supplementary Figure S2E,F.

Beta diversity was assessed using unweighted and weighted UniFrac and Bray–Curtis distances. The unweighted UniFrac distance revealed significant differences between all three groups: TB vs. PTB (q = 0.009), TB vs. CAOS (q = 0.012), and PTB vs. CAOS (q = 0.026). The weighted UniFrac distance showed a significant difference between the TB and PTB groups (q = 0.006), whereas other comparisons were not significant (q = 0.465). Similarly, the Bray–Curtis distance indicated a significant difference between the TB and PTB groups (q = 0.006), but not in other comparisons (Figure 6C–E). Homogeneity of dispersion (PERMDISP) confirmed no significant differences in group dispersion for any distance metric.

Among the placental microbiota, *Ureaplasma urealyticum* was relatively abundant in the PTB group, whereas *Ureaplasma parvum* showed higher relative abundance in the CAOS group. *Metamycoplasma hominis* was the most abundant in the PTB group. *Gardnerella vaginalis* exhibited a relatively high abundance in both the PTB and TB groups (Figure 5E–H). Other specific bacterial species are presented in Supplementary Figure S4.

## 4. Discussion

For the oral microbiota, alpha and beta diversity analyses revealed no notable differences in richness, evenness or overall community structure among the groups. Previous reports have shown similar results, with no significant group-wise differences in alpha or beta diversity [29,30]. Vidmar et al., note increases in *Veillonella, Prevotella*, and *Capnocytophaga* in PTB [29]. Similarly, Saadaoui et al. reported that *Prevotella*, *Alloprevotella*, *Mollicutes*, and *Prevotella enoeca* are more frequently detected in PTB [36]. While specific bacterial genera have been detected in PTB, the findings vary across studies, and the overall differences in microbial diversity appear to be limited. These findings suggest that differences in the structure of oral microbiota may not have a significant impact on perinatal complications. However, because this study did not include clinical periodontal assessments, the results may vary depending on the presence or severity of periodontal disease.

Regarding the vaginal microbiota, no significant differences were observed in alpha or beta diversity. Taxonomic composition in each group showed that the TB group exhibited a microbiota dominated by *Lactobacillus crispatus* and *Lactobacillus gasseri*, which is consistent with the stable and typical profile previously described by Huang et al., who reported an association between *L. crispatus* and a reduced risk of PTB [37]. In contrast, the PTB group showed an increased abundance of *Ureaplasma parvum* and *Gardnerella vaginalis*, suggesting an alteration in the vaginal microbial composition. The presence of *Ureaplasma*, *Mycoplasma*, and *Gardnerella* significantly associated with an increased PTB risk [27,38]. These findings are consistent with our results and support the link between PTB and vaginal dysbiosis. In the CAOS group, *Lactobacillus* was noticeably decreased, whereas a diverse range of anaerobic bacteria, such as *Gardnerella vaginalis*, *Prevotella bivia*, *Megasphaera*, *Sneathia amnii*, and *Atopobium vaginae* were dominant. The alpha diversity of the vaginal microbiota in pregnant women is significantly lower than that in non-pregnant women [39], and that higher diversity is associated with reduced protection by lactobacilli and an increased PTB risk [27,40]. These findings suggest that the vaginal microbiota in CAOS may be characterized by an unstable and dysbiotic state, which contributes to the disease.

Regarding the placental microbiota, significant differences in beta diversity were detected among the three groups, indicating variations in the microbial community structure. Unweighted UniFrac distance analysis, which considers the microbial lineages are present or absent in each community, revealed significant differences among the three groups. This suggests that differences in the presence or absence of specific taxa, rather than in the difference in bacterial abundance, may have contributed to the observed distinctions. This study evaluated the relative abundance of specific bacterial species that have been highlighted as being associated with PTB in the placental microbiota, and found that *Ureaplasma urealyticum*, *Metamycoplasma hominis*, *Gardnerella vaginalis*, *Bacteroides*, *Prevotella*, and *Porphyromonas* showed higher abundance in the PTB group. Prior research has shown that the placental microbiota of healthy term pregnancies resembles the oral microbiota, whereas that of PTB more closely resembles the vaginal microbiota [36]. These findings are consistent with the hypothesis that an ascending infection from the vaginal tract may be a major route of intrauterine infection in patients with PTB. As mentioned earlier, in uncomplicated TB, the vaginal microbiota is typically dominated by *Lactobacillus*, whereas adverse outcomes such as miscarriage or PTB are often associated with *Gardnerella vaginalis*, *Ureaplasma*, and other anaerobes. In this study, *Prevotella*, *Porphyromonas*, and *Bacteroides* were detected in the placentas of the PTB group. These bacteria are normally commensals of the oral cavity or gastrointestinal tract, and their presence in the placenta may indicate ectopic colonization and pathogenicity. Their transmission to the placenta may occur via hematogenous routes, highlighting the need for further research into the mechanisms of bacterial translocation.

This study suggests that, similar to PTB, CAOS may also be associated with placental microbiota differences and a potential shift in the vaginal microbiota. In future large-scale studies, microbial profiling may contribute to risk stratification and the development of preventive and therapeutic strategies for preterm birth, including CAOS.

In one CAOS case, *Ureaplasma* accounted for 19% of the vaginal microbiota and 98% of the placental microbiota (Supplementary Figure S4). *Ureaplasma species* are frequently detected in clinical CAM in term pregnancies without CAOS [41], and CAM is reportedly present in more than half of the CAOS cases [20,42]. Although the etiology of CAOS remains unclear, the detection of potentially pathogenic bacteria in both the vaginal and placental compartments in this study suggests that ascending infection may have contributed to its development. In addition to the disruption of the vaginal environment, whether selective overgrowth of these high-risk bacterial species contributes to disease onset warrants further investigation.

Although no significant differences in alpha or beta diversity were detected in the vaginal microbiota, differences in the average detection rates of specific bacteria were observed. These may not have reached significance because of substantial within-group variability and a limited sample size. Furthermore, because all participants were high-risk patients hospitalized for preterm labor, the alpha diversity may have been higher than that in typical pregnant populations.

Some placental samples were obtained after vaginal delivery (TB, 2/10; PTB, 5/7; CAOS, 0/3), which could potentially introduce vaginal contamination. However, the placenta was carefully sampled from the inner parenchyma under sterile conditions, and contamination through the sampling route was considered unlikely based on the detected bacterial profiles. Although the existence of a placental microbiota remains debated, negative control showed extremely low read counts. These findings suggest that the bacterial DNA detected in placental samples more likely reflects true biological colonization rather than contamination. Nevertheless, minimal contamination cannot be completely excluded, which remains an inherent limitation of low-biomass microbiome studies.

To the best of our knowledge, this study is the first to analyze the microbiota associated with CAOS using 16S rRNA sequencing. These results may play an important role in the investigation of the mechanisms of CAOS. Additionally, few studies have simultaneously evaluated the microbiota from multiple anatomical sites concerning perinatal complications. The use of a homogeneous cohort of Japanese women also minimized genetic and environmental heterogeneity.

This study had some limitations. First, the sample size was extremely small, and statistical power may have been insufficient to detect subtle differences in diversity or taxonomic composition. The observed differences in microbial composition, particularly at the taxon level, should be regarded as exploratory. To validate these preliminary observations, multicenter studies with larger sample sizes will be essential. Second, sampling was conducted only once during hospitalization, resulting in variability in gestational age at the time of sampling and potentially failing to capture dynamic changes in the microbiota throughout pregnancy. To generalize these findings to broader populations, validation through larger, longitudinal prospective cohort studies will be needed. Third, several clinical and environmental confounders—such as antibiotic administration, corticosteroid use, sexual activity, genital bleeding, dietary habits, socioeconomic status, and lifestyle—may influence microbiota composition. Although some of these factors were recorded, the limited sample size did not allow for statistical adjustment. However, as the study population was recruited from a single tertiary university hospital in Japan, access to healthcare and general socioeconomic backgrounds were likely relatively homogeneous, and the influence of these factors may have been limited. Fourth, we did not perform clinical periodontal examinations, which is a limitation because periodontal disease has been associated with PTB. Future studies should combine microbiota analysis with clinical periodontal assessments. Finally, because the microbiota varies by race and ethnicity, caution is warranted when generalizing these findings to other populations. Therefore, validation through future multicenter studies conducted in ethnically diverse populations will be essential to confirm these findings.

Despite these limitations, our study provides insights into the microbiota of the oral cavity, vagina, and placenta in high-risk pregnancies, and suggests potential microbial differences associated with PTB and CAOS. Further large-scale longitudinal studies are warranted to better understand the potential role of the microbiota in perinatal complications.

## 5. Conclusions

This study characterized oral, vaginal, and placental microbiota in pregnancies with threatened preterm labor. These findings suggest that the placental microbiota differs between TB and both PTB and CAOS groups, and that a potential shift in the vaginal microbiota may be involved in CAOS as well as in PTB. These observations suggest a possible microbial contribution to pathogenesis.

## Figures and Tables

**Table 1:** Comparison of maternal characteristics and perinatal outcomes among Term birth, Preterm birth, and CAOS groups.

Characteristic	N	TB, N = 10 ^1^	PTB, N = 7 ^1^	CAOS, N = 3 ^1^	*p*-Value ^2^
Age (year)	20	37.5 (30–42)	33.0 (29–35)	35.0 (32–36)	<0.05
Ethnicity	20				-
Japanese		10 (100%)	7 (100%)	3 (100%)	
Body mass index (kg/m^2^)	20	21.7 (17.8–25.6)	20.0 (17.6–24.2)	24.6 (24.3–24.8)	0.111
Nulliparas	20	7 (70%)	5 (71%)	2 (67%)	1.000
Mode of Delivery	20				
Vaginal Delivery		2 (20%)	5 (71%)	0 (0%)	0.072
Elective cesarean section		8 (80%)	0 (0%)	0 (0%)	<0.05
Emergent cesarean section		0 (0%)	2 (29%)	3 (100%)	<0.05
Gestational ages at vaginal sampling (week)	20	31.0 (24–33)	27.5 (24–33)	26.0 (23–27)	0.179
Gestational ages at placental sampling (week)	20	37.0 (34–37)	32.0 (25–35)	26.0 (23–28)	<0.05
Gestational ages at oral sampling (week)	20	31.0 (24–32)	29.0 (23–33)	26.0 (23–27)	0.245
Birth weight (g)	20	2780.0 (2006–3503)	2008.0 (724–2402)	808.0 (374–834)	<0.05
Apgar score (1 min)	20	7.5 (6–8)	5.0 (4–8)	3.0 (2–7)	0.062
Apgar score (5 min)	20	8.5 (7–9)	7.0 (6–9)	8.0 (5–8)	0.087
Umbilical artery pH	20	7.3 (7.22–7.38)	7.3 (7.26–7.43)	7.34 (7.19–7.38)	0.938

^1^ Median (IQR); *n* (%), ^2^ Kruskal–Wallis rank sum test; Fisher–Freeman–Halton exact test. TB: term birth group. PTB: preterm birth group. CAOS: chronic abruption oligohydramnios sequence group.

## Data Availability

The sequence raw data obtained using 16S rRNA sequencing analysis has been deposited to the Sequence Read Archive in the DNA Data Bank of Japan (DDBJ/DRA) (https://www.ddbj.nig.ac.jp/dra, accessed on 1 November 2025) under submission ID: PRJDB37520.

## Supplementary Materials

The following supporting information can be downloaded at: https://www.mdpi.com/article/10.3390/medicina61122096/s1, Figure S1: Alpha rarefaction curve; Figure S2: Alpha diversity analyses; Figure S3: Vaginal and placental microbial community structures in the CAOS group; Figure S4: Relative abundance of specific preterm-associated bacteria.

## Abbreviations

The following abbreviations are used in this manuscript:

PTB	Preterm Birth
TB	Term Birth
CAOS	Chronic Abruption-Oligohydramnios Sequence
CAM	Chorioamnionitis
PCR	Polymerase Chain Reaction
ASVs	Alignment of obtained amplicon Sequence Variants

## Appendix A

### Appendix A.1. Genomic DNA Extraction, and 16S rDNA Metagenomic Sequencing Library Preparation

DNA was extracted using the MagNA Pure 24 system (Pathogen 1000 hp 3.1 protocol) and eluted in 50 μL. Sterile distilled water, processed in the same manner as the samples, was used as a negative control. The V1–V2 region of the 16S rRNA gene was amplified using primers 27Fmod(5′-AGRGTTTGATYMTGGCTCAG-3′) and 338R(5′-CATGCTGCCTCCCGTAGGAGT-3′) with overhang adaptors for Illumina sequencing. PCR used Yeast-made Taq polymerase (Mitsui Chemicals Inc., Tokyo, Japan). DNA concentration was measured using DropSense16 to confirm extraction success. As bacterial loads were assumed low, samples proceeded without dilution.

A second PCR was performed to add index sequences based on the Nextera XT Index Kit v2 (Illumina). KAPA HiFi Polymerase (KAPA BIOSYSTEMS) was used, and the number of cycles was minimized to reduce amplification bias. The indexed PCR products were purified again using AMPure XP beads and used as sequencing libraries.

### Appendix A.2. Illumina Sequencing

Library concentrations for each sample were measured using Qubit (Life Technologies). The libraries were then pooled in equimolar amounts and sequenced on the Illumina MiSeq platform using 251 bp paired-end reads. Sequencing data were processed using MiSeq Reporter to generate fastq files for downstream analysis.

### Appendix A.3. Data Analysis

Microbiota analysis were performed with QIIME 2-2021.11 [34]. Low quality sequences were eliminated by the q2-demux plugin and denoising was carried out with DADA2 [43]. Alignment of obtained amplicon sequence variants (ASVs) were carried out using mafft [44]. Phylogeny tree was constructed fasttree2 [45]. Comparison of TB, PTB and CAOS groups usingα-diversity and β-diversity and Principal Coordinate analysis (PCoA) were carried out after samples were rarefied to 30,000 sequences, 25,000 sequences, and 2000 sequences per sample for oral samples, vaginal samples, and placental samples, respectively. These thresholds were determined based on the depth at which the alpha-rarefaction curves for each matrix reached a plateau. Taxonomy was assigned to ASVs using the q2-feature-classifier [46] classify-sklearn nai˙˙ve Bayes taxonomy classifier against the SILVA 138 [47].
